# A Statistical Model for Assessing Genetic Susceptibility as a Risk Factor in Multifactorial Diseases: Lessons from Occupational Asthma

**DOI:** 10.1289/ehp.8870

**Published:** 2006-11-13

**Authors:** Eugene Demchuk, Berran Yucesoy, Victor J. Johnson, Michael Andrew, Ainsley Weston, Dori R. Germolec, Christopher T. De Rosa, Michael I. Luster

**Affiliations:** 1 Division of Toxicology and Environmental Medicine, Agency for Toxic Substances and Disease Registry, Centers for Disease Control and Prevention, Atlanta, Georgia, USA; 2 Toxicology and Molecular Biology Branch and; 3 Biostatistics and Epidemiology Branch, Health Effects Laboratory Division, National Institute for Occupational Safety and Health, Centers for Disease Control and Prevention, Morgantown, West Virginia, USA; 4 Toxicology Operations Branch, Environmental Toxicology Program, National Institute of Environmental Health Sciences, National Institutes of Health, Department of Health and Human Services, Research Triangle Park, North Carolina, USA

**Keywords:** asthma, genetics, polygenic diseases, risk assessment, susceptibility genes

## Abstract

**Background:**

Incorporating the influence of genetic variation in the risk assessment process is often considered, but no generalized approach exists. Many common human diseases such as asthma, cancer, and cardiovascular disease are complex in nature, as they are influenced variably by environmental, physiologic, and genetic factors. The genetic components most responsible for differences in individual disease risk are thought to be DNA variants (polymorphisms) that influence the expression or function of mediators involved in the pathological processes.

**Objective:**

The purpose of this study was to estimate the combinatorial contribution of multiple genetic variants to disease risk.

**Methods:**

We used a logistic regression model to help estimate the joint contribution that multiple genetic variants would have on disease risk. This model was developed using data collected from molecular epidemiology studies of allergic asthma that examined variants in 16 susceptibility genes.

**Results:**

Based on the product of single gene variant odds ratios, the risk of developing asthma was assigned to genotype profiles, and the frequency of each profile was estimated for the general population. Our model predicts that multiple disease variants broaden the risk distribution, facilitating the identification of susceptible populations. This model also allows for incorporation of exposure information as an independent variable, which will be important for risk variants associated with specific exposures.

**Conclusion:**

The present model provided an opportunity to estimate the relative change in risk associated with multiple genetic variants. This will facilitate identification of susceptible populations and help provide a framework to model the genetic contribution in probabilistic risk assessment.

Common diseases of a chronic inflammatory nature such as asthma, Alzheimer disease, and cardiovascular disease are complex in nature, as they are variably influenced by genetic inheritance as well as environmental, physical, and lifestyle factors. Although genetic variants and their interactions probably define most interindividual variability in common disease susceptibility related to genetics (Moore 2003; Newton-Cheh and Hirschhorn 2005), they generally possess low or incomplete penetrance and consequently show low-risk associations in epidemiologic studies [e.g., odds ratios (ORs) ~ 1.5–2] (Hirschhorn et al. 2002; Lohmueller et al. 2003). Thus, for genetic variants to significantly affect disease severity or incidence, they must act cumulatively. Applying the composite genetic contribution to the risk assessment process would allow for identifying the most genetically susceptible groups in the population. In light of this, a multiplicative gene–gene interaction model was developed to allow for estimating the combinatorial contribution of multiple genetic variants to disease risk. To illustrate the utility of this model, asthma was selected as an example of a common multifactorial disease as the pathological processes have been well established and a number of genetic variants that influence the disease have been identified in association studies. Data were compiled from 14 genetic association studies linking 16 susceptibility variants in inflammatory, immune, and chemical metabolism genes to the risk of developing disease. Our model predicts that a broad heterogeneity exists in the population disease risk defined by genetic variation. The broadened risk profile is amenable, however, to segregating the population by relative risk level, which should allow for identification of the most susceptible populations. The current limitations and assumptions of this approach, which include lack of joint distributions, limited information on epistasis and the influence of other potential variables, such as exposure, are discussed.

## Materials and Methods

### Study design

Population-based genetic association studies deal with relatively small effects against a complex background. Therefore, association studies are often statistically under-powered and poorly standardized. General concerns include a lack of attention to sampling and study design, inconsistent criteria for clinical assessment, population stratification, the use of genetic markers that are only modestly correlated with disease, and publication bias. Considering these concerns, we extracted data from a public database (PubMed 2004) using the terms “asthma,” “polymorphism,” and “gene.” We included studies that followed standard asthma diagnosis criteria (physician-diagnosed asthma), used case–control study design, and described associations with *p*-values < 0.05 in the analyses to help limit potential false positive associations. The genetic variants we selected were not intended to be an exhaustive list of published variants of candidate genes that have been associated with asthma but rather representative of those in which significant associations have been repeatedly observed, known to cause changes in protein expression, and act through established pathways for allergic response (Blumenthal 2005; Malerba and Pignatti 2005). As reflected in the published literature, most of the variants included in the analyses are associated with increased risk for developing asthma rather than decreased risk. Hence, we included only one variant that is considered protective.

Although published genetic association studies have used a variety of methods for presenting results, we selected disease-associated variant genotypes as opposed to allele frequencies, as the relationship of the latter to disease has not been clearly defined. Most of the genes and chromosomal regions that have been associated with disease are linked to chromosomes 5q, 11q, 12q, and 6p. We stratified candidate genes into three groupings based on their role in the pathogenesis of asthma. The first group (12 variants) included genes related to inflammation and immune cascades known to be involved in allergic asthma, such as the interleukin 4 (*IL-4)* receptor variant R567. The second group consisted of atopy-associated gene variants contained within the human leukocyte antigen (*HLA)* class II family. The third grouping consisted of variants associated with chemical metabolism, represented by the *N*-acetyltransferase (*NAT)* polymorphism associated with slow acetylation. The genes and variants used in the analyses are presented in Table 1.

### Statistical model

We modeled the single-gene variants listed in Table 1 as binary outcomes and generated polygenotypes from single-gene genotypes using a recursive binomial scheme. Under this scheme all possible permutations of single-gene polymorphisms are considered, and the total number of polygenic profiles is 2*n*, where *n* is the number of genes used in the analysis (sixteen in the present study). We estimated the frequencies of the genotype profiles from single-gene frequencies as a product of epidemiologically derived single-gene frequencies. Susceptibility to disease was expressed in terms of ORs. Polygenetic ORs were calculated from single-gene ORs under the assumption of genetic independence (absence of linkage disequilibrium); that is, for each variant, the enrichment or depletion of cases with that variant does not affect the frequency of any other variant. Therefore, single-gene frequencies multiply to estimate the frequency of polygenotypes. The model we proposed also assumes that the selected genes are biologically independent and thus, no epistasis at the level of protein function is considered. Thus, we used a logistic regression model without interaction cross-terms. This results in a multiplicative OR for a polygenotype in which the combinatorial genotype OR is generated simply by multiplying individual ORs for the variants that are present for a specific genotype profile.

## Results

**Figure 1:**
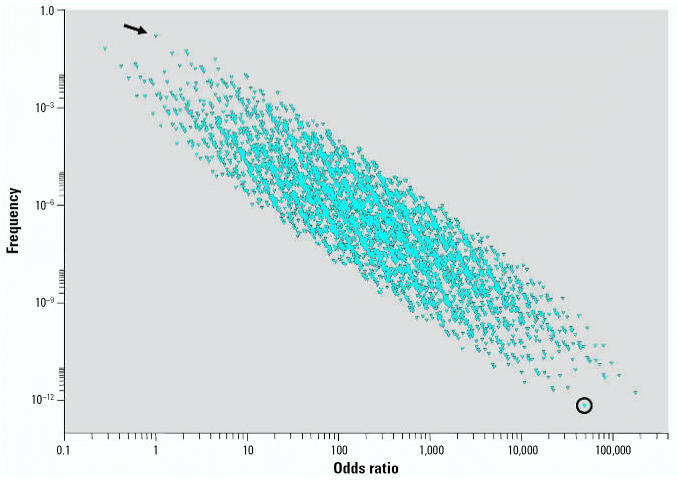
Frequencies and ORs of genotypes in a control population calculated using 16 gene variants listed in Table 1. Each point represents a unique genotype combination. Referent genotype profile is identified by the arrow (OR = 1). Genotypic profile composed of all minor variants is identified by the circle.

ORs obtained from 16 genetic variants reported to be associated with allergic asthma were used to estimate the contribution of genetic variation in disease risk. Each possible genotype in the population was assigned a categorical binary variable representing either the wild-type (0) or the variant (minor) genotype (X) identified from each of the selected studies. Thus, each possible combination can be represented as a 16-dimensional profile where, for instance, {XXXXXXXXXXXXXXXX} denotes a genotype profile that contains only minor variants. We obtained the frequency for each profile from the reported frequencies in each original study (Table 1). Control frequencies from each study were reported to be consistent with those found in the general population with similar ethnicities. Figure 1 summarizes the relationship between the frequency of each of the 65,536 (2^16^) potential genotypic profiles and risk of developing allergic asthma under the described model and illustrates the concept that susceptibility variants can shift the risk distribution to the right or left depending upon whether the variant has an adverse or protective role, respectively. The various genotype profiles represented in Figure 1 are enriched with those genotypes that increase the risk of asthma, thus accounting for the right-sided skew in the scat-terplot. The arrow in this diagram indicates the location of the wild-type genotype profile {OOOOOOOOOOOOOOOO} with its associated OR of 1. It is evident that the frequency and magnitude of risk are highly correlated, such that very high-risk genotypes are exceedingly rare in the population and, in fact, the highest risk polygenotype is so rare that it is unlikely to even exist. The genotypes that have an OR < 1 are due to the inclusion of the protective –627 polymorphism in the interleukin 10 (*IL-10)* gene (Hang et al. 2003), which reduces the overall risk for developing asthma. The right-sided skew shown in Figure 1 is consistent with current evidence that the vast majority of identified variants have been associated with an adverse rather than protective contribution (Ober and Hoffjan 2006). It is not known whether these variants are evolutionarily driven or because adverse variants are more actively studied and identified than those that are protective.

**Figure 2:**
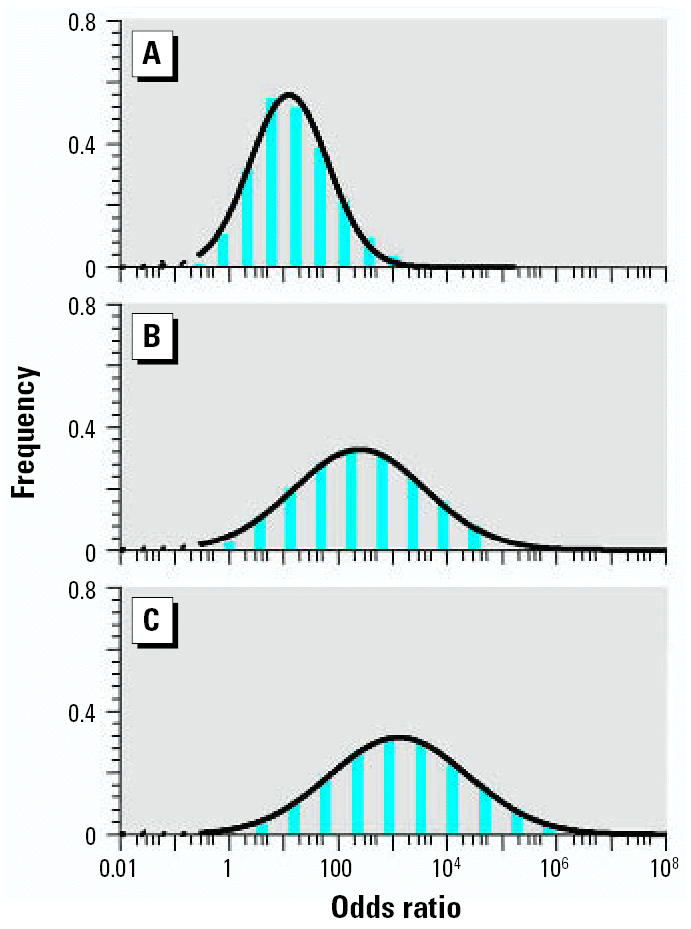
Distribution of relative disease risk calculated using asthma-associated gene variants grouped by their biological attribution: (*A*) 12 group I variants only; (*B*) with three group II variants added to *A*; (*C*) with group III variant added to *B*.

Examination of a single susceptibility gene can separate the study population into only two risk groups, those with and those without the mutation. In contrast, modeling the impact of multiple disease variants associated with immune and inflammatory mediators of allergic asthma (group 1 variants) provides a pseudo-continuous log-normal relative disease risk distribution in the population (Figure 2A). Inclusion of variants associated with atopy (Figure 2B) and acetylation rate (Figure 2C) further shifts the distribution toward the higher risk. Equally evident is the impact of combining variants on the standard deviation of disease risk in the population. As we added more disease variants to the model, the risk distribution broadened, allowing better distinction of the population into high and low risk categories. The frequencies associated with such risk levels will be important in defining susceptible populations that need increased protection with respect to exposure, as well as for risk management.

**Figure 3:**
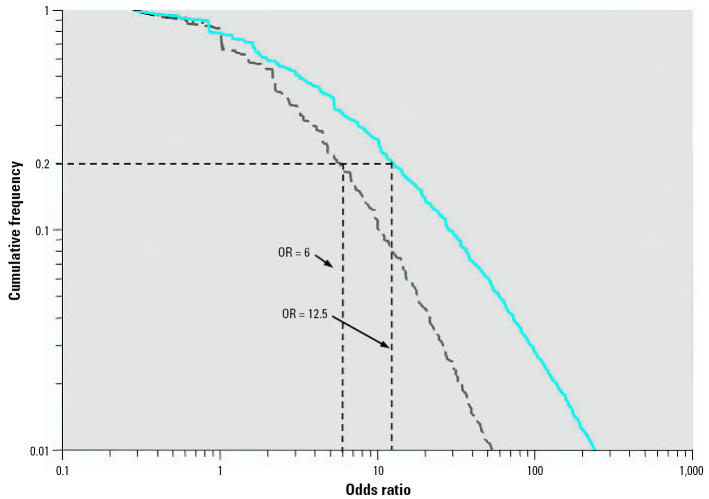
The low end of cumulative distribution of ORs calculated using asthma-associated genetic variants (Table 1). The dashed gray line corresponds group I variants; the solid blue line represents risk distribution following addition of the group III variant.

The present model provided an opportunity to quantify the relative change in risk associated with the presence of genetic variants in the general population. This is exemplified in Figure 3 where the dashed gray line represents the risk profile for the most common genotypes modeled from the 12 asthma susceptibility genes (group 1 variants) and the solid blue line shows the risk profile when the *NAT1* variant is added. These curves indicate that in individuals carrying the *NAT1* mutation, the risk of asthma increases approximately 2-fold or more in 20% of the possible polygenotypes present in a population of workers exposed to diisocyanates. Acetylation rate is thought to affect the metabolism of diisocyanates, which in turn correlates with differences in diisocyanates-induced asthma rates (Wikman et al. 2002). If only those variants common to allergens (first group) are considered, one would estimate that 20% of the population would have at least 6-fold increase susceptibility relative to the referent genotype profile. Thus, this model allows for incorporation of exposure information as an independent variable, illustrating why variants such as those involved in atopy or chemical metabolism, would need to be included separately in identifying the number of individuals in a population at increased risk.

## Discussion

We used a logistic regression model to estimate the joint contribution of multiple genetic variants on the risk of developing allergic asthma. Allergic asthma data sets were used because disease prevalence is relatively high—estimated to be approximately 7.5% (range, 5.2–10.3%) among the U.S. population (Mannino et al. 2002)—and the pathological processes as well as many of the disease mediators have been identified (Barrios et al. 2006). The latter allowed for an additional level of confidence in that the genetic variants selected for modeling are associated with well-established pathological processes. Although data sets from other common polygenic diseases may have sufficed, such as Alzheimer or cardiovascular disease, their pathological processes are less well defined.

Single-genotype ORs provided by genetic association studies is the available input to model the polygenotype–disease association. ORs are functions of the logistic regression coefficients. Thus, the logistic regression model, which is commonly used in epidemiology studies, provides a straightforward approach for combining single genotype ORs to model the combinatorial genotype ORs (Kleinbaum and Klein 2002). However, the accuracy of this model to capture true polygenic susceptibility remains to be determined. Currently, our laboratory in conjunction with a National Institute for Occupational Safety and Health–funded multicenter asthma genotype program (RO1 OH008795-01) centered at the University of Cincinnati is collecting data on multiple variants in a single population to help establish the validity of this model.

A major limitation of using a multiplicative interaction model to derive polygenic risk from single-gene studies is that epistatic relationships are not considered. Although the model assumes there is no statistical interaction, it does not account for potential biological interactions at the protein level that may modify risk. For example, epistasis likely plays a role in determining complex phenotypes such as allergic asthma. However, epistatic relationships can be generated only from efforts to genotype functional variants in all potential target genes in a single population. This presents a potential problem because the population frequency of polygenotypes is generated from the product of single-gene frequencies, making complex polygenotypes very rare. Therefore, as the number of genes increases, the number of individuals required in order to estimate polygenic risk markedly increases, thereby necessitating the need for a modeling approach. This is especially true for occupational populations, given the low number of employees exposed to a given occupational allergen and the even lower incidence of disease. It is possible that the effects of epistasis in multifactorial diseases are relatively modest. For example, a recent epidemiologic study of breast cancer demonstrated that only 17% of three gene combinations showed statistical evidence of epistasis (Aston et al. 2005). More simple schemes to help define epistasis may involve interactions derived from genomic and proteomic data, which can allow for decoding transcriptional and posttranscriptional interaction networks (Johnson et al. 2004). As more reliable biological and epidemiologic information regarding joint effects and epistasis becomes available, new patterns of interaction can be added to the model, which will allow for more accurate risk estimates.

Genetic independence is another assumption when using this model. Linkage disequilibrium is the deviation from probabilistic independence between alleles at two different loci. This deviation from independence can have different causes, such as a lack of independent segregation or recombination, or any number of other evolutionary forces. Therefore, an association of a certain genetic marker with disease may reflect the etiologic role of the locus of interest but not of the marker itself. Since a multiplicative approach for the joint effects of genotypes between loci was assumed in this model, only the gene variants known not to be in linkage disequilibrium were considered.

The choice of mode of inheritance (allelic or genotypic) used for analyses can have a marked impact on risk estimates. Most genetic association studies reduce three genotypes to two by using recessive (assuming heterozygotes have no increased risk), co-dominant (a per-allele effect that places heterozygotes halfway between minor and major homozygous genotypes), or dominant genetic models (in which heterozygotes have the same increased risk as minor homozygous genotypes). However, some studies ignore the heterozygotes and compare only minor and major homozygous genotypes. Because the biological function of the variations is rarely known, it is difficult to determine the mode of inheritance. As indicated by Minelli et al. (2005), if the assumption of genetic model is in doubt, then the best approach would be to perform joint pair-wise comparison, that is, genotype associations. Therefore, using the disease-associated variant genotypes identified in the individual studies as opposed to decomposing the population into allele frequencies is an appropriate approach to capture and model the impact of multiple variants. As biological data regarding the inheritance modes of variants become available, a biologically justified strategy for incorporating each susceptibility variant can be applied.

In conclusion, the increased risk for developing a multifactorial disease based upon disease-susceptibility variants with moderate effects was estimated using a logistic regression model assuming multiplicative gene–gene interactions. Although limited by our current lack of knowledge regarding the role of gene–gene and gene–environment interactions in multifactorial common diseases, such a model, without interaction cross-terms, is the first step in the development of a comprehensive polygenic risk model. These types of analysis can provide information on the relative changes in risk associated with genetic variability found inherently in the population and help provide a framework to model the genetic contribution in probabilistic risk assessment. Such information may also provide opportunities for targeting preventative or therapeutic actions to high-risk populations. In a broader context, the polygenic model for genetic susceptibility contributes to the design of a virtual toxicology testing laboratory, which would help to reduce animal testing and adverse human exposures. With rapid advances in the identification of genetic variants in the population, underscored by the Human Genome and HapMap Projects (The International HapMap Consortium 2003; Pennisi 2001), advances in high throughput genotyping methodology and improved understanding of the molecular events involved in disease processes, key susceptibility polygenotypes driving risk for common complex diseases may be identified.

## Figures and Tables

**Table 1:** Genes related to immune/inflammatory processes and environmental/occupational exposures in asthma.

Gene (Entrez Gene ID)^a^	Variation	Frequency	OR (mean)	*p*-Value	Reference
Group I (immune, inflammatory)					
*TGF-*β (7040)	−509	0.117	2.456	0.0102	Silverman et al. 2004
*TLR-10* (81793)	2322	0.034	2.237	0.0235	Lazarus et al. 2004
*TNF-*α (7124)	−308	0.223	1.505	0.0444	Witte et al. 2002
*MCP-1* (6347)	−2518	0.089	2.703	0.0055	Szalai et al. 2001
*IL-13* (3596)	−1055	0.019	7.756	0.0081	van der Pouw Kraan et al. 1999
*CD-14* (929)	−159	0.098	3.143	0.0355	Woo et al. 2003
*IL-18* (3606)	105	0.109	1.830	0.0068	Higa et al. 2003
*IL-10* (3586)	−627	0.289	0.278	0.0222	Hang et al. 2003
*RANTES* (6352)	−28	0.219	2.233	0.0006	Yao et al. 2003
*IL-4R* (3566)	R576	0.018	8.185	0.0429	Rosa-Rosa et al. 1999
*ACE* (1636)	Ins/del	0.160	4.472	0.0018	Gao et al. 2000
*Fc*ɛ*RI*β (2206)	E237G	0.252	2.155	0.0003	Cui et al. 2003
Group II (atopy)					
*HLA-DQA1* (3117)	0301	0.081	8.774	0.0010	Aron et al. 1996
*HLA-DQB1* (3119)	0302	0.083	6.794	0.0039	Aron et al. 1996
*HLA-DRB1* (3123)	4	0.026	24.588	0.0023	Aron et al. 1996
Group III (metabolism)					
*NAT1* (9)	Slow/fast	0.250	8.625	0.0059	Wikman et al. 2002

Ins/del, insertion/deletion

a Gene loci and gene identification numbers are from Entrez Gene (2006).

## References

[b1-ehp0115-000231] Aron Y, Desmazes-Dufeu N, Matran R, Polla BS, Dusser D, Lockhart A (1996). Evidence of a strong, positive association between atopy and the HLA class II alleles DR4 and DR7. Clin Exp Allergy.

[b2-ehp0115-000231] Aston CE, Ralph DA, Lalo DP, Manjeshwar S, Gramling BA, DeFreese DC (2005). Oligogenic combinations associated with breast cancer risk in women under 53 years of age. Hum Genet.

[b3-ehp0115-000231] Barrios RJ, Kheradmand F, Batts L, Corry DB (2006). Asthma: pathology and pathophysiology. Arch Pathol Lab Med.

[b4-ehp0115-000231] Blumenthal MN (2005). The role of genetics in the development of asthma and atopy. Curr Opin Allergy Clin Immunol.

[b5-ehp0115-000231] Cui T, Wang L, Wu J, Xie J (2003). The association analysis of F_cɛ_RIβ with allergic asthma in a Chinese population. Chin Med J (Engl).

[b6-ehp0115-000231] Entrez Gene 2006. Entrez Gene Home Page. Bethesda, MD: National Center for Biotechnology Information. Available: http://www.ncbi.nlm.nih.gov/entrez/query.fcgi?DB=gene [accessed 11 August 2006].

[b7-ehp0115-000231] Gao J, Lin Y, Xiao Y, Xu K, Xu W, Zhu Y (2000). Polymorphism of angiotensin-converting enzyme gene and genetic susceptibility to asthma with familial aggregation. Chin Med Sci J.

[b8-ehp0115-000231] Hang LW, Hsia TC, Chen WC, Chen HY, Tsai JJ, Tsai FJ (2003). Interleukin-10 gene -627 allele variants, not interleukin-I beta gene and receptor antagonist gene polymorphisms, are associated with atopic bronchial asthma. J Clin Lab Anal.

[b9-ehp0115-000231] Higa S, Hirano T, Mayumi M, Hiraoka M, Ohshima Y, Nambu M (2003). Association between interleukin-18 gene polymorphism 105A/C and asthma. Clin Exp Allergy.

[b10-ehp0115-000231] Hirschhorn JN, Lohmueller K, Byrne E, Hirschhorn K (2002). A comprehensive review of genetic association studies. Genet Med.

[b11-ehp0115-000231] Johnson CD, Balagurunathan Y, Tadesse MG, Falahatpisheh MH, Brun M, Walker MK (2004). Unraveling gene-gene interactions regulated by ligands of the aryl hydrocarbon receptor. Environ Health Perspect.

[b12-ehp0115-000231] Kleinbaum DG Klein M 2002. Logistic Regression—A Self-Learning Text. New York:Springer-Verlag.

[b13-ehp0115-000231] Lazarus R, Raby BA, Lange C, Silverman EK, Kwiatkowski DJ, Vercelli D (2004). TOLL-like receptor 10 genetic variation is associated with asthma in two independent samples. Am J Respir Crit Care Med.

[b14-ehp0115-000231] Lohmueller KE, Pearce CL, Pike M, Lander ES, Hirschhorn JN (2003). Meta-analysis of genetic association studies supports a contribution of common variants to susceptibility to common disease. Nat Genet.

[b15-ehp0115-000231] Malerba G, Pignatti PF (2005). A review of asthma genetics: gene expression studies and recent candidates. J Appl Genet.

[b16-ehp0115-000231] Mannino DM, Homa DM, Akinbami LJ, Moorman JE, Gwynn C, Redd SC (2002). Surveillance for asthma—United States, 1980–1999. MMWR Surveill Summ.

[b17-ehp0115-000231] Minelli C, Thompson JR, Abrams KR, Thakkinstian A, Attia J (2005). The choice of a genetic model in the meta-analysis of molecular association studies. Int J Epidemiol.

[b18-ehp0115-000231] Moore JH (2003). The ubiquitous nature of epistasis in determining susceptibility to common human diseases. Hum Hered.

[b19-ehp0115-000231] Newton-Cheh C, Hirschhorn JN (2005). Genetic association studies of complex traits: design and analysis issues. Mutat Res.

[b20-ehp0115-000231] Ober C, Hoffjan S (2006). Asthma genetics 2006: the long and winding road to gene discovery. Genes Immun.

[b21-ehp0115-000231] Pennisi E (2001). What’s next for the genome centers?. Science.

[b22-ehp0115-000231] PubMed 2004. PubMed Home Page. Bethesda, MD:National Center for Biotechnology Information, National Library of Medicine, National Institutes of Health. Available: http://www.ncbi.nlm.nih.gov/entrez [accessed 10 December 2004]

[b23-ehp0115-000231] Rosa-Rosa L, Zimmermann N, Bernstein JA, Rothenberg ME, Khurana Hershey GK (1999). The R576 IL-4 receptor alpha allele correlates with asthma severity. J Allergy Clin Immunol.

[b24-ehp0115-000231] Silverman ES, Palmer LJ, Subramaniam V, Hallock A, Mathew S, Vallone J (2004). Transforming growth factor-beta1 promoter polymorphism C-509T is associated with asthma. Am J Respir Crit Care Med.

[b25-ehp0115-000231] Szalai C, Kozma GT, Nagy A, Bojszko A, Krikovszky D, Szabo T (2001). Polymorphism in the gene regulatory region of MCP-1 is associated with asthma susceptibility and severity. J Allergy Clin Immunol.

[b26-ehp0115-000231] The International HapMap Consortium (2003). The International HapMap Project. Nature.

[b27-ehp0115-000231] van der Pouw Kraan TC, van Veen A, Boeije LC, van Tuyl SA, de Groot ER, Stapel SO (1999). An IL-13 promoter polymorphism associated with increased risk of allergic asthma. Genes Immun.

[b28-ehp0115-000231] Wikman H, Piirila P, Rosenberg C, Luukkonen R, Kaaria K, Nordman H (2002). *N*-Acetyltransferase genotypes as modifiers of diisocyanate exposure-associated asthma risk. Pharmacogenetics.

[b29-ehp0115-000231] Witte JS, Palmer LJ, O’Connor RD, Hopkins PJ, Hall JM (2002). Relation between tumour necrosis factor polymorphism TNFalpha-308 and risk of asthma. Eur J Hum Genet.

[b30-ehp0115-000231] Woo JG, Assa’ad A, Heizer AB, Bernstein JA, Hershey GK (2003). The -159 C→T polymorphism of CD14 is associated with nonatopic asthma and food allergy. J Allergy Clin Immunol.

[b31-ehp0115-000231] Yao TC, Kuo ML, See LC, Chen LC, Yan DC, Ou LS (2003). The RANTES promoter polymorphism: a genetic risk factor for near-fatal asthma in Chinese children. J Allergy Clin Immunol.

