# Erratum

**Published:** 2007-05

Demchuk et al. have reported two errors in their article [ Environ Health Perspect 115:231–234 (2007)]. First, in the legend to Figure 1, the authors stated that the figure presented frequencies and odds ratios of “16 gene variants listed in Table 1.” However, only the first group of 12 genes in Table 1 was taken into consideration to generate the figure. This correction also requires a change on page 232 (20th line of “Results”) because fewer polygenotypes are possible with this combination of 12 genes than with 16 genes (65,536 polygenotypes). The corrected sentence is as follows:

Figure 1 summarizes the relationship between the frequency of each of the 4,096 (2^12^) potential genotypic profiles and risk of developing asthma under the described model and illustrates the concept that susceptibility variants can shift the risk distribution to the right or left depending upon whether the variant has an adverse or protective role, respectively.

Second, the frequency distributions shown in Figure 2 were mistakenly weighted by single nucleotide polymorphism (SNP) frequencies for the population of cases provided in each source study. Instead, the distributions should have been weighted by SNP frequencies from the controls in each source study, which approximate the SNP frequencies reported for the general population. The corrected figure appears below.

These errors were introduced when new figures were generated during the final revision of the paper. The authors emphasize that these changes do not alter the concepts that they addressed in their article.

The authors apologize for the errors.

## Figures and Tables

**Figure 2:**
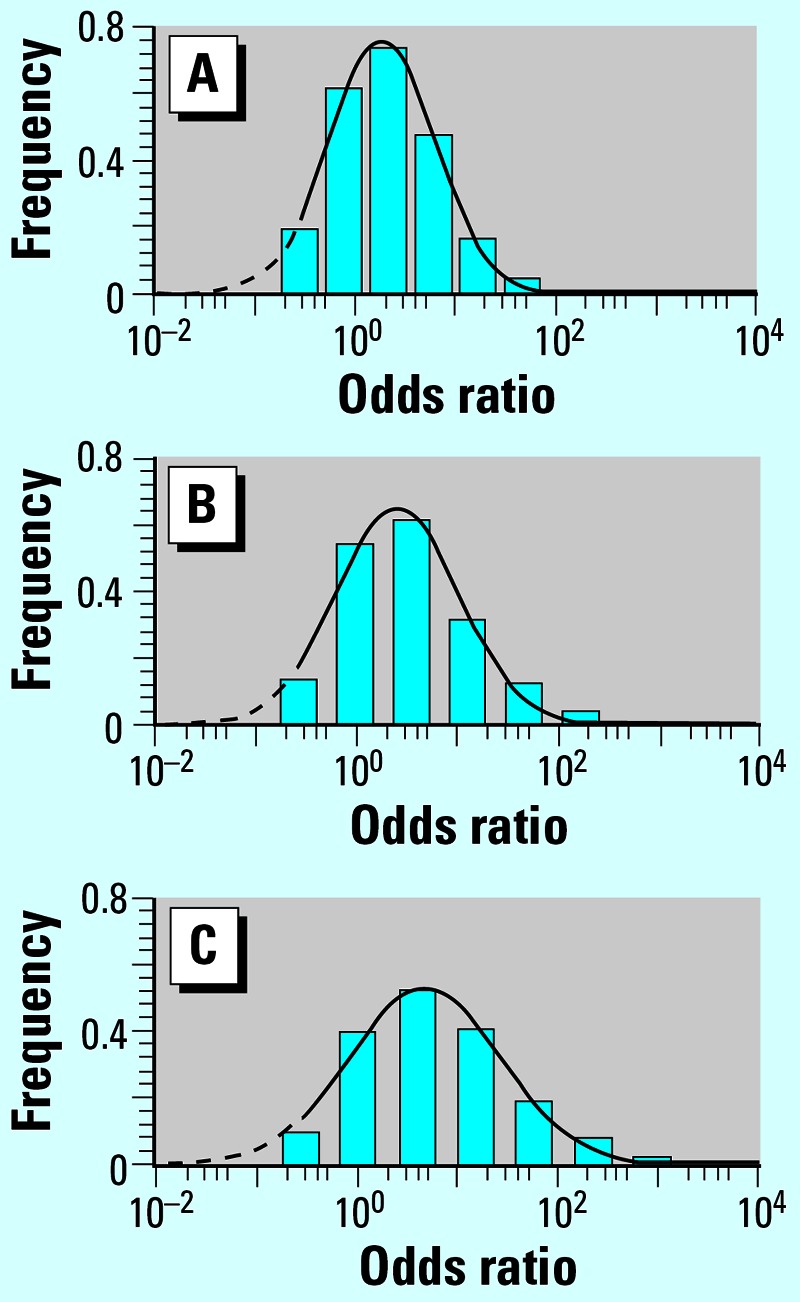
Distribution of relative disease risk calculated using asthma-associated gene variants grouped by their biological attribution: (*A*) 12 group I variants only; (*B*) with three group II variants added to (*A*); and (*C*) with group III variant added to (*B*).

